# Supplementary material for: Awareness and Knowledge Regarding the Consumption of Dietary Fiber and Its Relation to Self-Reported Health Status in an Adult Arab Population: A Cross-Sectional Study
Source: Int J Environ Res Public Health. 2020 Jun 13;17(12):4226. doi: 10.3390/ijerph17124226 (PMC7345011; doi:10.3390/ijerph17124226)
Supplement: Supplementary file 1 [file ijerph-17-04226-s001.pdf]

Awareness, Knowledge and Consumption of Dietary Fiber and its Relation with Self-reported Health Status in Adult Arab Population: A Cross-Sectional Study

Hanan Alfawaz[1,2,#,*], Nasiruddin Khan[3,#], Haya Alhuthayli[1], Kaiser Wani[2], Muneerah A. Aljumah[5], Malak Nawaz Khan Khattak[2], Saad A. Alghanim[4] and Nasser M. Al-Daghri[2].

Estimation of Cronbach's Alpha coefficient: The responses from 50 participants of the pilot study were used to calculate the Cronbach's alpha coefficient for four sections of the questionnaire related to knowledge, preference, perception and consumption of DF. This was done using SPSS and the results are displayed below

Section 1: Consumption of Food items rich In Dietary fibers (30 items)

Case Processing Summary

		N	%
Cases	Valid	41	82.0
	Excluded[a]	9	18.0
	Total	50	100.0

a. Listwise deletion based on all variables in the procedure.

Item-Total Statistics

	Scale Mean if Item Deleted	Scale Variance if Item Deleted	Corrected Item-Total Correlation	Cronbach's Alpha if Item Deleted
Salad	12.951	37.398	.157	.866
Cooked_vegetables	13.000	36.500	.326	.863
fruit_juice	13.244	37.939	-.007	.873
Fresh_peeled_Vegetables	13.366	37.538	.057	.871
Fruit_with_peel	13.244	37.939	-.007	.873
Brown_Juice	13.098	36.090	.344	.863
Cereals_Such_Wheat	13.146	35.578	.417	.861
Maize	13.512	35.306	.467	.860
Burghul	13.561	34.402	.673	.854
Barley	13.683	36.972	.240	.865
Millet	13.756	37.139	.291	.864

Quinoa	13.683	35.422	.609	.857
Peanuts	13.317	34.822	.514	.858
pistachios	13.049	35.698	.454	.860
Almonds	13.220	34.076	.663	.854
Walnuts	13.488	34.856	.540	.858
Pineapple_Dried	13.390	35.294	.435	.860
Dried_Dates	13.293	37.812	.013	.872
Hazelnut	13.512	35.556	.421	.861
Raisin	13.415	35.399	.421	.861
Figs	13.463	34.355	.621	.855
Apricot	13.561	35.352	.486	.859
Shea_Seeds	13.585	35.599	.454	.860
Beans_Fava	13.122	35.560	.432	.861
Oats	13.244	35.289	.440	.860
Lentils_Yellow	13.268	35.901	.331	.863
Chickpeas	13.244	34.139	.645	.854
Green_Beans	13.366	34.638	.547	.857
White_Beans	13.683	36.672	.310	.864
Libya_beans	13.585	35.049	.565	.857

Reliability Statistics

Cronbach's Alpha	N of Items
.866	30

Scale Statistics

Mean	Variance	Std. Deviation	N of Items
13.829	38.145	6.1762	30

Section 2: Knowledge about role of DF in health (8 items)

Case Processing Summary

		N	%
Cases	Valid	32	64.0
	Excluded[a]	18	36.0
	Total	50	100.0

a. Listwise deletion based on all variables in the procedure.

Item-Total Statistics

	Scale Mean if Item Deleted	Scale Variance if Item Deleted	Corrected Item-Total Correlation	Cronbach's Alpha if Item Deleted
Help_Prevent_Obesity	11.4688	7.612	.565	.657
Fiber_Regulated_Blood_Suger	11.1875	7.383	.338	.705
Reduce_Blood_Cholestrol	11.2188	6.951	.526	.654
Prevention_of_bowel	11.1563	7.555	.298	.715
Increase_Problem_Constipation	10.7500	8.452	.319	.700
Too_much_fiber_harmful	10.6250	7.661	.466	.672
Fiber_Flatulence	10.9375	7.996	.358	.693
Fiber_CVD	11.4688	7.870	.473	.673

Reliability Statistics

Cronbach's Alpha	N of Items
.712	8

Scale Statistics

Mean	Variance	Std. Deviation	N of Items
12.6875	9.641	3.10502	8

Section 3: Preference of foods while eating out (6 items)

Case Processing Summary

		N	%
Cases	Valid	32	64.0
	Excluded[a]	18	36.0
	Total	50	100.0

a. Listwise deletion based on all variables in the procedure.

Reliability Statistics

Cronbach's Alpha	N of Items
.774	6

Item-Total Statistics

	Scale Mean if Item Deleted	Scale Variance if Item Deleted	Corrected Item-Total Correlation	Cronbach's Alpha if Item Deleted
FastFood_Peeledfruits	9.0625	2.060	.383	.778
FastFood_brownbread	9.0000	2.129	.390	.772
FastFood_whitebread	8.9375	2.060	.585	.729
FastFood_friedpotato	9.0000	1.871	.654	.706
FastFood_legumes	9.0000	1.935	.585	.724
FastFood_cookedveg	9.0625	1.867	.563	.729

Scale Statistics

Mean	Variance	Std. Deviation	N of Items
10.8125	2.738	1.65466	6

Section 4: Perceptions towards foods rich in DF's (4 items)

Case Processing Summary

		N	%
Cases	Valid	29	58.0
	Excluded[a]	21	42.0
	Total	50	100.0

a. Listwise deletion based on all variables in the procedure.

Reliability Statistics

Cronbach's Alpha	N of Items
.779	4

Item-Total Statistics

	Scale Mean if Item Deleted	Scale Variance if Item Deleted	Corrected Item-Total Correlation	Cronbach's Alpha if Item Deleted
High_Price	5.3103	1.793	.645	.692

Do_Not_Like_Taste	5.6552	1.948	.562	.736
Limited_Health_Benefits	5.3448	1.805	.578	.729
Not_easily_Available	5.7241	1.921	.549	.742

Scale Statistics

Mean	Variance	Std. Deviation	N of Items
7.3448	3.091	1.75816	4
